# The Hospital Treatment of Diseases of Heart and Lungs

**Published:** 1883-02

**Authors:** 


**The Hospital Treatment of Diseases of Heart and Lungs**; with over 350 Formulæ and Prescriptions, as examplified in the Hospitals of New York City. By CHARLES H. GOODWIN, M. D. New York: Published by the Author, 255 West 53d Street, New York City. Price, $1.50.

This volume of about 200 pages contains a resume of the treatment of the several diseases of the lungs and heart, as exemplified in the services of the leading physicians of New York City, connected with Bellevue, Roosevelt, St. Luke's, New York, Presbyterian, German, Charity, St. Francis and Mt. Sinai Hospitals. It is a decided improvement upon the numerous books of formulæ, which are as useless as they are numerous. This book has a real value, and it is interesting to compare the various modes of treatment adopted at the various hospitals. Under the head of "Pneumonia," for instance, the author gives the general treatment followed at "Bellevue" in the respective services of Flint, Loomis, Alonzo Clark, Thompson and Janeway—that of Dr. Draper, at the New York; Post and Smith, at the Presbyterian; Beverly Robinson, at "Charity," and Leaming, at St. Luke's. We would recommend the book as well worth buying.

